# Role of Hydrogen Concentration in Strength and Damage of Polycrystalline Iron Under Triaxial Tension

**DOI:** 10.3390/ma19040673

**Published:** 2026-02-10

**Authors:** Yi Liao, Runting Chen, Wanghui Li, Xia Tian, Taolong Xu, Kun Wang, Jun Chen, Meizhen Xiang

**Affiliations:** 1 School of Mechanical Engineering, Southwest Petroleum University, Chengdu 610500, China; 2 Institute of High Performance Computing (IHPC), Agency for Science, Technology and Research (A∗STAR), 1 Fusionopolis Way, #16-16 Connexis, Singapore 138632, Singapore; 3 College of Mechanics and Engineering Science, Hohai University, Nanjing 210098, China; 4 Petroleum Engineering School, Southwest Petroleum University, Chengdu 610500, China; 5 State Key Laboratory of Cemented Carbide, College of Materials Science and Engineering, Hunan University, Changsha 410082, China; 6 National Key Laboratory of Computational Physics, Institute of Applied Physics and Computational Mathematics, Beijing 100088, China

**Keywords:** molecular dynamic simulation, polycrystalline iron, triaxial tension, hydrogen atoms, dislocations

## Abstract

The mechanical response of the iron–hydrogen (Fe-H) system under triaxial tensile loading is systematically investigated using molecular dynamics simulations. The study focuses on how hydrogen concentration affects the stress state and void evolution and further explores its coupled effects with temperature. The results indicate that when the hydrogen concentration is less than or equal to 1%, hydrogen atoms impede dislocation motion, thereby retarding void growth by promoting dislocation entanglement and the formation of loop structures. Moreover, the evolution of void volume exhibits a typical three-stage characteristic: an initial slow growth phase, a rapid growth phase, and a decelerated growth phase after coalescence. In addition, the evolution of void surface area in the model essentially results from competition between two mechanisms: the decrease caused by void collapse and coalescence and the increase caused by void expansion. Cluster configuration analysis reveals that void formation around the clusters serves as a critical turning point for their structural stability, and the subsequent evolution of the voids leads to a substantial reduction in local structural stability. The analysis of the coupling effect between temperature and hydrogen concentration reveals that under high-temperature conditions, temperature plays a key role in determining the strength, while the strengthening effect of low hydrogen concentrations can be neglected. Additionally, at low temperatures, hydrogen concentration has a negligible effect on structure, but under elevated temperatures, increased hydrogen concentration markedly intensifies the degree of structural disorder.

## 1. Introduction

As the most widely used structural material in modern industry, steel possesses reliable mechanical properties, which are critical for ensuring the safe operational performance of engineering systems [1,2]. However, in complex service environments, steel often faces severe challenges, with hydrogen embrittlement (HE) being a particular problem [3,4,5,6]. HE is a phenomenon in which hydrogen atoms can penetrate a metallic material, resulting in a significant deterioration of the material’s mechanical properties. Several theories have been proposed to explain this phenomenon, including the hydrogen-enhanced local plasticity (HELP) theory [7,8], hydrogen-enhanced decohesion (HEDE) mechanism [9,10,11], and adsorption-induced dislocation emission (AIDE) theory [12,13]. From an atomic-scale perspective, hydrogen atoms, due to their extremely small atomic radius, exhibit high mobility within metal lattices [14]. More importantly, hydrogen atoms tend to accumulate at microscopic defects such as grain boundaries (GBs), dislocations, and second phases, thereby forming localized hydrogen-enriched zones [15,16,17,18,19], which not only lead to localized stress concentration but also reduce the interfacial bonding strength, thus exacerbating hydrogen embrittlement behavior. Current research indicates that hydrogen embrittlement susceptibility is influenced by multiple factors, including grain size, temperature, hydrogen concentration, and strain rate [20,21,22,23,24,25]. Among these, the interaction between hydrogen and grain boundaries is considered the critical factor determining HE behavior [20]. Therefore, in-depth investigations into how hydrogen–grain boundary interactions influence the mechanical properties of metallic materials and the underlying mechanisms involved in the hydrogen embrittlement process have become a key focus of current research.

In recent years, significant progress has been made in the study of HE mechanisms, and the application of a variety of advanced characterization techniques has provided new research paradigms for uncovering hydrogen–material interaction mechanisms. Li et al. [26] have revealed how pre-torsional deformation-induced gradient structures affect HE susceptibility in pure iron. Zhang et al. [27] have found that the presence of martensite profoundly increases the hydrogen diffusion depth and material susceptibility to HE. Lee et al. [28] have systematically investigated the fracture behavior of polycrystalline iron foil and demonstrated that hydrogen atoms significantly accelerate crack propagation. Mitomi et al. [29] have examined the effect of grain size on the hydrogen embrittlement behavior of pure iron. Lee et al. [30] have systematically studied the hydrogen embrittlement behavior of iron during the electrochemical hydrogen charging process. Lublińska et al. [31] have revealed the crystallographic characteristics of hydrogen-induced damage by hydrogen charging experiments on polycrystalline and single-crystal ferritic stainless steels.

To address the limitations of conventional characterization techniques, which make it difficult to observe the dynamic behavior of hydrogen atoms in real time, atomic-scale computational simulations have emerged as a crucial tool for investigating hydrogen embrittlement mechanisms in recent years. Static electronic structure calculations based on first principles provide fundamental theoretical support for hydrogen–material interactions. Ito et al. [32] have revealed that hydrogen in bcc-Fe preferentially occupies octahedral sites. Huang et al. [33] and Mai et al. [34] have shown through density functional theory calculations that Σ5 grain boundaries exhibit higher hydrogen solubility and diffusion rates than Σ3 grain boundaries while significantly weakening grain boundary cohesion. Li et al. [35] have found that hydrogen atoms occupy octahedral interstitial sites, inducing phase transitions in tension and eventually leading to intergranular brittle fracture.

Building upon static electronic structure studies, molecular dynamics (MD) simulations can further elucidate the evolutionary mechanisms of hydrogen embrittlement from a kinetic perspective. Liu et al. [36] have conducted MD simulations to examine hydrogen segregation behavior in the Mg-H system and its effects on the fracture properties. The results indicate that hydrogen atoms preferentially segregate at grain boundaries and that the critical energy release rates of both the matrix and grain boundaries decrease substantially with increasing hydrogen concentration. Zheng et al. [37] have analyzed the effects of hydrogen segregation and grain boundary coupling on the mechanical properties of nickel. The study revealed that hydrogen promotes dislocation nucleation at high-energy grain boundaries while suppressing dislocation activity at low-energy grain boundaries. Li et al. [38] have used MD simulations to elucidate how the hydrogen concentration gradient regulates the crack extension mode in α-Fe. Zhou et al. [39] explored the mechanical response of nanocrystalline iron during uniaxial tensile stretching at different hydrogen concentrations and grain sizes, and the results demonstrate that grain refinement can significantly enhance the hydrogen embrittlement resistance of the material. Wan et al. [40] have simulated the effect of hydrogen atoms on the mechanical behavior of grain boundaries in α-Fe and discovered that hydrogen segregation promotes the grain boundary activation and triggers stress concentration, which in turn accelerates grain boundary decohesion. Matsumoto et al. [41] have revealed the mechanism of how hydrogen concentration affects dislocation behavior in BCC iron through MD simulations, finding that hydrogen atoms induce a significant pinning effect at dislocation lines, and this effect becomes more pronounced with increasing hydrogen concentrations. Xing et al. [42] determined that hydrogen atoms affect intergranular fracture in α-Fe through MD simulations. Their study found that hydrogen atoms promote phase transformation and intergranular fracture by reducing the surface energy and increasing local stress, ultimately releasing the stress and energy at the grain boundaries.

Although extensive research has been devoted to elucidating the microscopic mechanisms of hydrogen-induced damage, the hydrogen-induced damage behavior of materials becomes more complex under high-strain-rate conditions. More critically, departing from the common assumption in most existing models that hydrogen atoms are randomly distributed in metal materials, our study constructs a model with hydrogen segregation at grain boundaries through heat treatment, better reflecting the physical characteristics of hydrogen accumulation at grain boundaries in practical scenarios. The present study establishes a triaxial tensile model of polycrystalline iron under hydrogen-doped conditions using MD simulations to systematically investigate the evolution of the mechanical properties of polycrystalline materials with different hydrogen concentrations. Furthermore, the study elucidates the coupling effect of hydrogen concentration and temperature on the strength of polycrystalline iron under triaxial tension. Our research aims to reveal atomic-scale mechanistic evolution laws rather than establish macroscopic statistical constitutive models. The findings not only contribute to a deeper understanding of hydrogen-induced damage mechanisms but also provide important theoretical support and design guidance for protecting metallic materials against hydrogen damage in complex service environments. The structure of this paper is as follows: Section 2 introduces the modeling methodology and analytical procedure. Section 3 discusses the influence of hydrogen concentration on the strength and damage behavior of the Fe-H system and analyzes the coupled effect of temperature and hydrogen concentration on the mechanical properties of the system. Finally, conclusions are given in Section 4.

## 2. Simulation Methods and Modeling

First, a polycrystalline α-Fe model is built using ATOMSK (Version 0.13) [43]. The model dimensions are 171.3 Å × 171.3 Å × 171.3 Å, with the x, y, and z coordinates aligned along the [100], [010], [001] crystallographic directions, respectively. The interaction forces between hydrogen and iron atoms are described using the embedded atom (EAM) potential [44], which has been employed in studies involving triaxial tensile simulations [45]. MD simulations are conducted using the LAMMPS software (2022 version, developed by Sandia National Laboratories, Albuquerque, NM, USA) [46]. During the simulation, periodic boundary conditions are imposed in the x, y, and z directions, and the time step is set to 1 fs.

**Figure 1:**
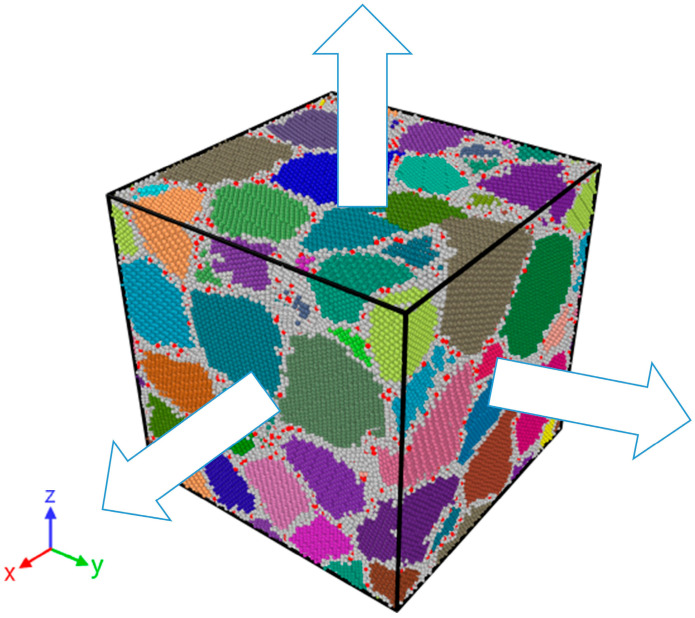
Initial model configuration: hydrogen-segregated model.

Subsequently, hydrogen atoms are introduced into the model to construct polycrystalline models with varying hydrogen concentrations of 0.5%, 1%, 3%, and 5%. In this study, *C_H_* (hydrogen concentration) is expressed as the atomic percentage of hydrogen relative to the total number of atoms, with the selection of hydrogen concentrations based on Reference [47]. In this paper, the hydrogen-segregated model is selected for simulation, with the model containing 3% hydrogen concentration taken as an example (see Figure 1 for the initial configuration, where different colors represent distinct grains, white regions denote grain boundaries, hydrogen atoms are marked in red, and the arrow indicates the direction of stretching). Hydrogen atoms are randomly added to the model, which is then heat-treated [48]. The model is first heated from 300 K to 700 K at a heating rate of 800 K/ns. Afterward, the model is maintained at 700 K for 1.5 ns to reach a more stable structure. Subsequently, the system is cooled down from 700 K to 300 K at the same cooling rate of 800 K/ns, resulting in a polycrystalline model with hydrogen atoms segregated at grain boundaries.

In the initial stage, the model temperatures are controlled, with zero pressure, followed by energy minimization. Next, the system is then fully relaxed for 100 ps under the NPT ensemble to ensure it reached an equilibrium state. After relaxation, the temperature is controlled using the velocity rescaling method, and a triaxial tensile simulation is performed under the NVT ensemble by applying equal strain rates along the x, y, and z coordinate axes. When considering temperature effects, the temperatures are set to 300 K, 600 K, 900 K, and 1200 K. The strain rate is set at 5 × 10^9^ s^−1^, based on References [49,50]. When temperature is not specifically mentioned in the paper, a default value of 300 K was used.

Post-processing in this study was performed using the visualization capabilities of OVITO software (Version 3.12) [51]. Local atomic structures were identified through Common Neighbor Analysis (CNA). Voids were detected, and their volume fraction and surface area were calculated using the “Construct Surface Mesh” modifier. Dislocations were analyzed with the DXA modifier, and atomic stress tensors were computed based on the Virial theorem.

## 3. Results and Discussion

### 3.1. Influence of Hydrogen Concentration on Strength and Damage

**Figure 2:**
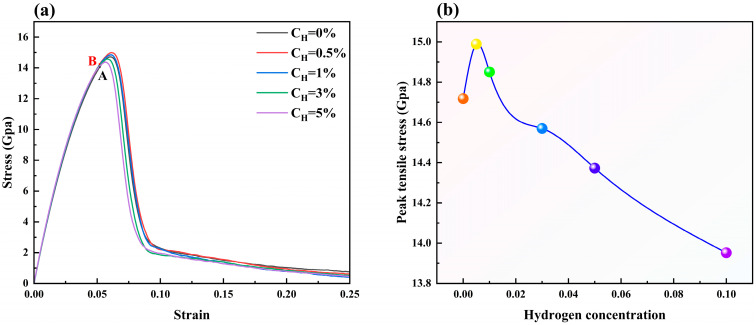
Stress–strain diagrams for different hydrogen concentrations: (**a**) stress variation diagrams, wherein Points A and B correspond to the states at a strain of 0.52 with hydrogen concentrations of 0% and 0.5%, respectively; (**b**) maximum tensile stress variation with hydrogen concentration.

Figure 2a illustrates the variation in tensile stress after the introduction of different concentrations of hydrogen atoms into the polycrystalline model, while Figure 2b further reveals the trend of the peak tensile stress with hydrogen concentration. As can be seen from Figure 2, the peak tensile stress of the model increases rapidly in the initial stage with increasing hydrogen concentration, subsequently transitioning to a progressive decline. At low concentrations, this phenomenon is consistent with the conclusion of Reference [52], that is, that stress intensity increases with the increase in hydrogen atom concentration. Specifically, the peak tensile stress reaches a maximum of 14.99 GPa at a 0.5% hydrogen concentration and decreases to 14.37 GPa at 5%.

**Figure 3:**
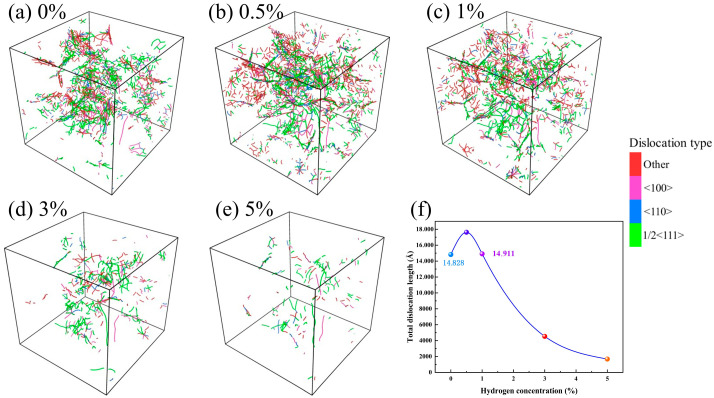
Dislocation distribution at different hydrogen concentrations at the initial moment of stretching (just as the relaxation completes): (**a**) *C_H_* = 0%, (**b**) *C_H_* = 0.5%, (**c**) *C_H_* = 1%, (**d**) *C_H_* = 3%, and (**e**) *C_H_* = 5%; (**f**) illustrates the variation in dislocation length with hydrogen concentration.

**Figure 4:**
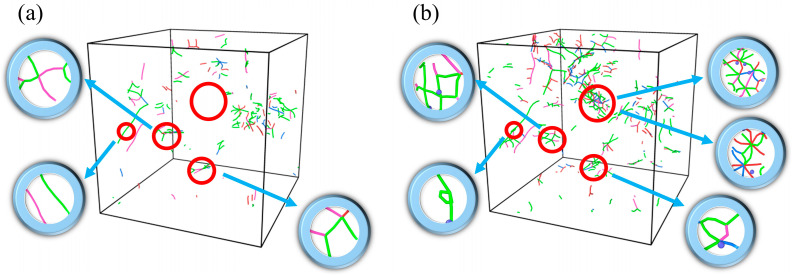
Dislocation distributions at different hydrogen concentrations during triaxial tensile: (**a**) *C_H_* = 0% and (**b**) *C_H_* = 0.5%.

To investigate the reason for the increase in peak stress at a hydrogen concentration of 0.5%, the dislocation line distribution is analyzed for different hydrogen concentration conditions at the initial moment of stretching (just as the relaxation completes), as shown in Figure 3. The analysis demonstrates that the dislocation type is dominated by 1/2<111>, and the number and length of dislocations tends to increase and then decrease with increasing hydrogen concentration. At a hydrogen concentration of 0.5%, the dislocations form a more complex interwoven and extended structure within the cubic lattice, exhibiting higher-density dislocation networks. This feature indicates that in this system, the dislocation density in the solid solution is higher and the pinning effect is augmented, thus restricting dislocation slip. Given that Figure 3 only shows a slightly higher dislocation density at a 0.5% hydrogen concentration compared to the hydrogen-free condition, it is necessary to further analyze the evolution of dislocations during the tensile process. To this end, Figure 4 presents the distribution of dislocation lines and their local details during triaxial tensile under two sets of different hydrogen concentration conditions (blue atoms represent hydrogen atoms in the magnified views). The stress states correspond to the characteristic points A and B in Figure 2a, respectively. At a hydrogen concentration of 0.5%, both dislocation density and length within the model increase remarkably. Notably, in the region marked by red circles, the action of hydrogen atoms causes the dislocations to entangle with each other and form a looped structure, which significantly impedes dislocation motion [53], and the increase in dislocation resistance can limit the rate at which voids grow [54], thereby remarkably increasing the tensile strength of the material. Our simulations reveal a transition in the failure mechanism dominated by hydrogen concentration. At low concentrations (*C_H_* ≤ 1%), hydrogen-dislocation interactions (pinning effects related to the HELP mechanism) play the dominant role. However, in our simulations, the shielding effect typically associated with the HELP mechanism does not appear; instead, strengthening is observed. This is because the shielding effect requires hydrogen atoms to keep up with the motion of dislocations. In molecular dynamics simulations, the strain rate is extremely high and dislocations move very rapidly; the diffusion of hydrogen atoms is far too slow to keep pace with dislocation motion and form a “shielding cloud” (as required by the quasi-steady state of HELP). Consequently, hydrogen atoms act as static obstacles, generating a pinning effect on the fast-moving dislocations rather than a softening effect due to shielding. As the concentration increases, the HEDE mechanism gradually overcomes dislocation effects, with hydrogen-induced decohesion at grain boundaries leading to premature void nucleation and a reduction in overall strength.

### 3.2. Void Evolution

**Figure 5:**
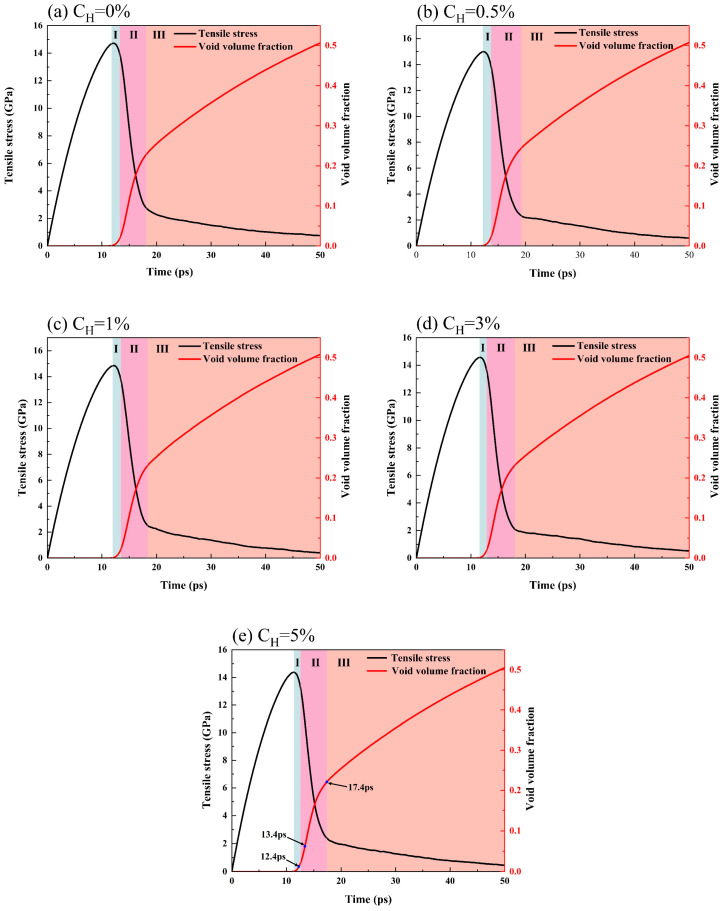
Relationship between tensile stress and void volume fraction for different hydrogen concentrations: (**a**) *C_H_* = 0%, (**b**) *C_H_* = 0.5%, (**c**) *C_H_* = 1%, (**d**) *C_H_* = 3%, and (**e**) *C_H_* = 5%. (I) denotes the initial growth stage, (II) the rapid growth phase, and (III) the slow growth stage.

The formation of voids during the triaxial tensile process is inevitable; therefore, analyzing their evolution is key to understanding the material’s damage behavior. In order to thoroughly investigate the influence of hydrogen on the material’s damage evolution, the evolution characteristics of tensile stress and the void volume fraction under varying hydrogen concentration conditions are further analyzed, as presented in Figure 5. The three vertical lines in the figure mark key time points: the first line indicates the onset of void nucleation, corresponding to the void nucleation time in Table 1; the second line marks the moment when the void volume fraction grows to 2%; and the third line indicates the point at which the void volume fraction begins to stabilize. According to Figure 5 and Table 1, with the increase in hydrogen concentration, the void nucleation time exhibits an initial delay followed by subsequent advancement. In particular, at a 5% hydrogen concentration, void formation occurs 0.4 ps earlier compared to the hydrogen-free condition. This demonstrates that an appropriate hydrogen content (0.5%) can delay damage initiation, whereas excessive hydrogen concentrations can exacerbate the damage, making hydrogen-induced failure more likely.

It can be further observed from Figure 5 that the evolution of the void volume fraction can be divided into three stages. (1) During initial growth, the tensile stress curve reaches the peak value, the voids start to nucleate, and the void volume fraction grows slowly. (2) In the rapid growth phase, when the void volume fraction increases to 2%, it begins to increase rapidly while the tensile stress decreases dramatically. This trend indicates that the accelerated growth of the void volume fraction directly leads to the sharp stress attenuation. (3) In the slow growth stage, the growth rate of the voids gradually slows down, and the stress reduction rate decreases significantly.

**Figure 6:**
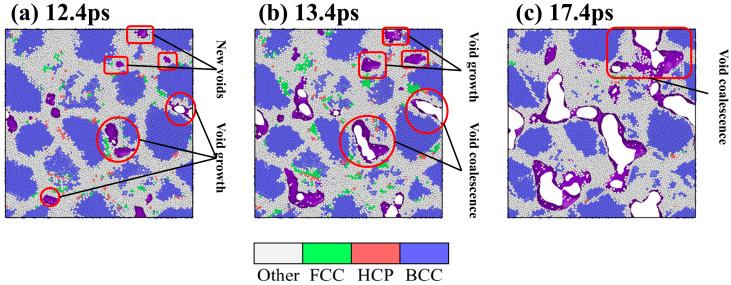
Evolution of voids at a hydrogen concentration of 5%: (**a**) 12.4 ps, (**b**) 13.4 ps, and (**c**) 17.4 ps.

To thoroughly elucidate the microscopic mechanism of void evolution, the evolution of the material’s internal structure is visualized and analyzed. As shown in Figure 6, the atomic structure is identified using the CNA method, in which the purple area represents the void boundary. Taking the 5% hydrogen concentration model as an example (corresponding to the three time points in Figure 5e), the complete process of void nucleation, growth and coalescence is dynamically demonstrated. It is obvious that void nucleation, growth and coalescence primarily occur within grain boundary regions.

**Figure 7:**
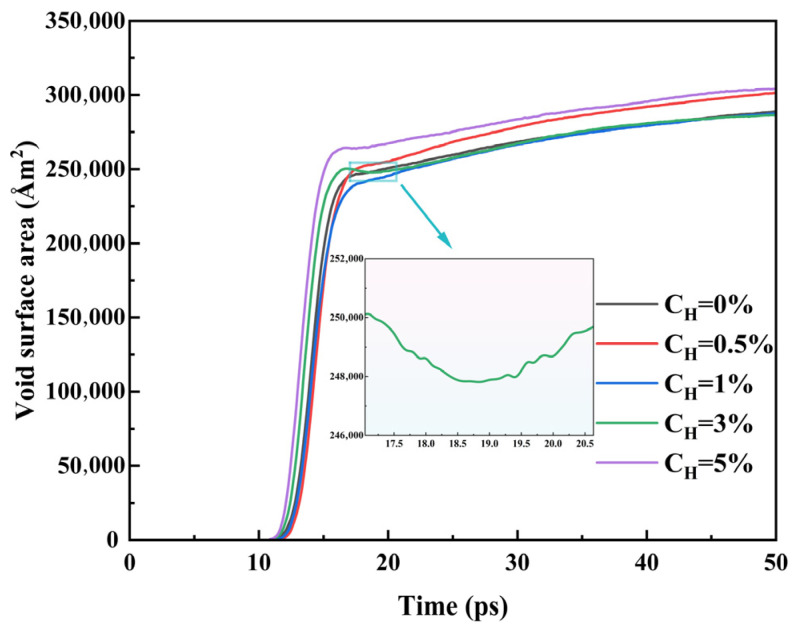
Evolution of void surface area over time at different hydrogen concentrations.

Considering the combined effects of multiple factors such as the morphological diversity of voids, coalescence patterns, and growth modes, the growth trends of both void volume and surface area may not be entirely consistent. Therefore, it is necessary to further analyze the temporal evolution characteristics of the void surface area. Figure 7 presents the temporal evolution curve of the void surface area under different hydrogen concentration conditions. The results demonstrate that the void surface area exhibits an overall increasing trend with time. Specifically, during the rapid-growth stage of void surface area, the growth rate is relatively slow at lower hydrogen concentrations (0.5%, 1%), whereas it shows a significant increase under higher hydrogen concentrations.

**Figure 8:**
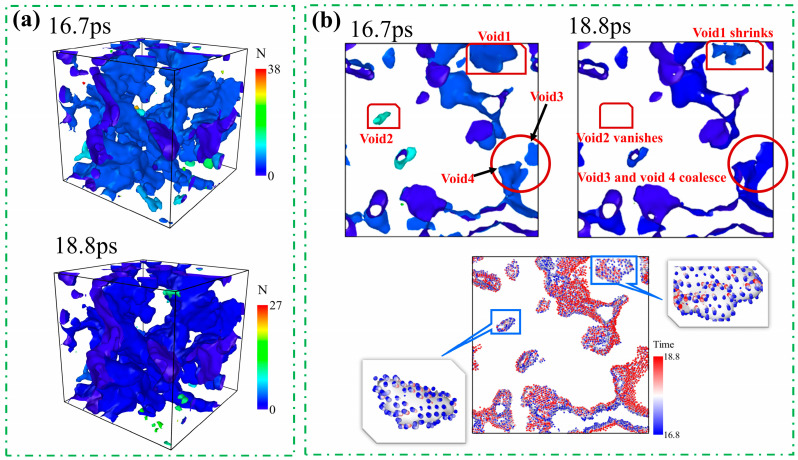
Void collapse and coalescence mechanisms at a hydrogen concentration of 3% (time in ps): (**a**) shows the void diagrams at 16.7 ps and 18.8 ps; (**b**) displays the cross-sectional views from 16.7 ps to 18.8 ps.

Interestingly, at a hydrogen concentration of 3%, the void surface area shows a transient decrease over a certain period (see localized enlargement in Figure 7). To explore the microscopic mechanism underlying this phenomenon, Figure 8 employs atomic trajectory tracking to analyze the morphological evolution of voids from 16.7 ps to 18.8 ps. As can be seen from Figure 8a, the number of voids decreases significantly within this time interval, which may result from either the collapse of individual voids or the coalescence of adjacent voids. Figure 8b presents the void distribution at two instants (16.7 ps and 18.8 ps) and illustrates their dynamic behavior based on atomic motion trajectories (the color bar represents time). It can be seen that void 1 contracts inward, void 2 vanishes completely, and void 3 and void 4 merge. All these processes lead to a reduction in the surface area of the voids. Meanwhile, analysis of the atomic trajectories shows that some voids remain in an expanding state, which contributes to an increase in void surface area. Thus, the evolution of void surface area in the model essentially results from the competition between two mechanisms: the decrease caused by void collapse and coalescence and the increase caused by void expansion. Consequently, the transient decline in void surface area observed in Figure 7 during the period from 16.7 ps to 18.8 ps is precisely due to the dominance of the collapse and coalescence mechanisms within this time interval.

**Figure 9:**
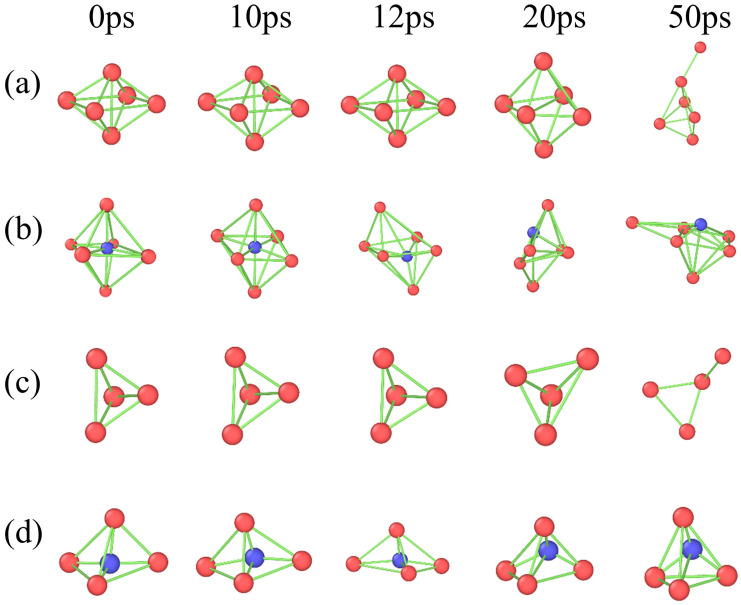
Schematic of cluster structure evolution over time, with blue representing hydrogen atoms, red representing iron atoms and the green regions represent the bonding connections between atoms: (**a**) Octahedral configuration without hydrogen, (**b**) Octahedral configuration with hydrogen, (**c**) Tetrahedral configuration without hydrogen, (**d**) Tetrahedral configuration with hydrogen.

**Figure 10:**
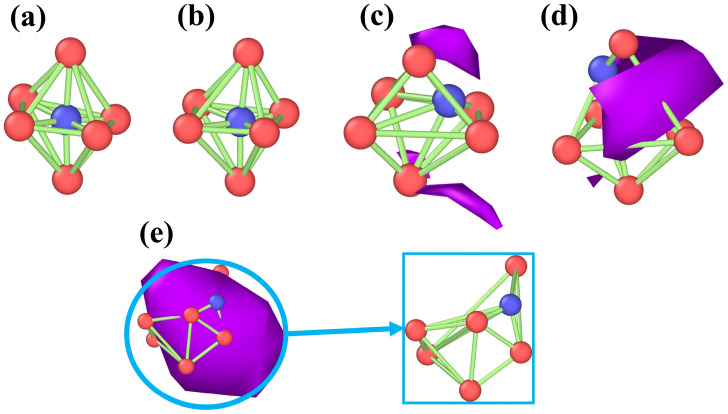
The evolution of cluster configurations caused by void formation, with blue representing hydrogen atoms, red representing iron atoms, and the purple area representing the void. (**a**,**b**) correspond to the state prior to void nucleation; (**c**) represents the stage of incipient void nucleation around the clusters; (**d**,**e**) denote the configurational changes of the clusters during void growth.

Figure 9 shows the evolution of cluster configurations at different time points for a hydrogen concentration of 1% (where 12 ps represents the void formation moment). The configurations are presented from top to bottom as octahedral without hydrogen atoms, octahedral with hydrogen atoms, tetrahedral without hydrogen atoms, and tetrahedral with hydrogen atoms. In the polycrystalline iron model, hydrogen atoms, owing to their small atomic radius, typically occupy tetrahedral and octahedral interstitial sites [55]. As can be seen from Figure 9, at the initial stage (0 ps), both tetrahedral and octahedral configurations maintain stability, with hydrogen atoms occupying interstitial sites of the crystal lattices and no significant migration tendencies. As the stretching process proceeds (0 ps to 10 ps), the tetrahedral and octahedral cluster configurations gradually undergo a slight deformation, and the introduction of hydrogen atoms leads to a minor adjustment in the interatomic bonds between iron atoms, an increase in the spacing of some of the atoms, and a slight breakdown of the local symmetry. From 12 ps to 20 ps, the nucleation and growth of voids occur around hydrogen-containing tetrahedral and octahedral cluster configurations, causing significant distortion in these hydrogen-bearing clusters and consequently destabilizing the local atomic structure. During this period, no void formation occurs around the hydrogen-free cluster configurations; therefore, these cluster structures remain stable. It is worth noting that at 50 ps, all cluster configurations undergo significant changes, which is directly correlated with the interconnection of internal voids. To visually illustrate the influence of the void formation process on the stability of the cluster structure, Figure 10 illustrates the evolution of the octahedral configuration under hydrogen-containing conditions, in which red spheres represent iron atoms, blue spheres denote hydrogen atoms, and the purple regions correspond to the void. Figure 10a,b show relatively intact octahedral structures before void formation. When the voids begin to form, local atomic coordination is disrupted, leading to changes in cluster configuration. As the voids expand further, the cluster structure gradually undergoes significant distortion. This set of results demonstrates that void formation around the clusters serves as a critical turning point for their structural stability, and the subsequent evolution of the voids leads to a substantial reduction in local structural stability.

### 3.3. Hydrogen–Temperature Effect

**Figure 11:**
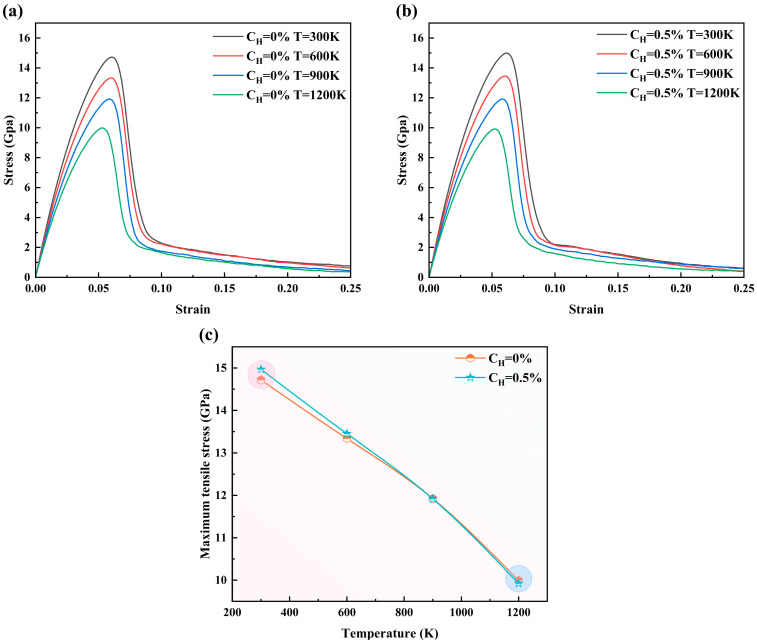
The stress–strain curves at temperatures ranging from 300 K to 1200 K: (**a**) *C_H_* = 0%, (**b**) *C_H_* = 0.5%, (**c**) represents the variation of peak tensile stress with temperature.

The above sections primarily investigate the effects of hydrogen atoms on the mechanical properties of the material. This section investigates the influence of the coupling between T (temperature) and hydrogen concentration on the strength of the Fe-H system. Figure 11a,b depicts the stress–strain curves of models with hydrogen concentrations of 0% and 0.5% at different temperatures, while Figure 11c further presents the variation trend of peak tensile stress with temperature. From Figure 11a,b, it can be observed that under certain hydrogen concentration conditions, the thermal softening effect is pronounced. Figure 11c clearly shows that at 300 K, the introduction of a small number of H atoms (*C_H_* = 0.5%) results in a higher peak tensile stress compared to the pure iron model (as indicated by the pink circle in Figure 11c, demonstrating that the strengthening effect of hydrogen atoms is evident, which is consistent with the conclusion stated earlier. Nonetheless, the peak tensile stress of the hydrogen-containing model becomes almost identical to that of the pure-iron model as the temperature increases to 900 K and 1200 K (as shown by the blue circles in Figure 11c). This phenomenon indicates that under high-temperature conditions, temperature plays a key role in determining strength, while the strengthening effect of hydrogen can be neglected. In practice, hydrogen atoms can serve as alloying solute atoms in the design of high-performance materials, such as austenitic stainless steels, to enhance the material’s strength [56].

**Figure 12:**
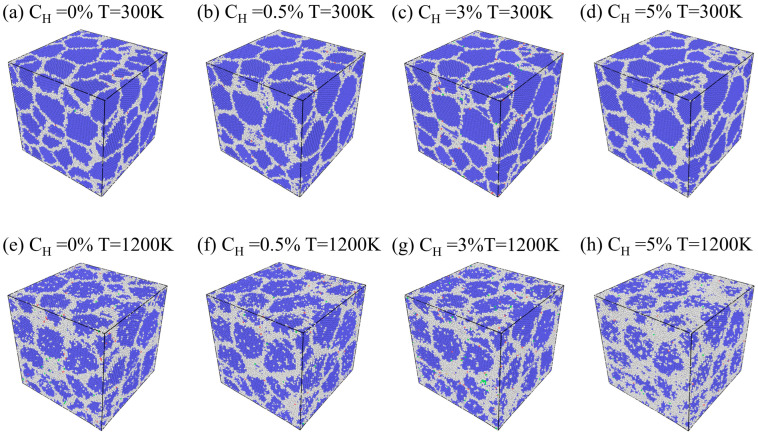
Internal atomic structure at different hydrogen concentrations and temperatures: from left to right, hydrogen concentrations are *C_H_* = 0%, 0.5%, 3%, and 5%; from top to bottom, temperatures are *T* = 300 K and 1200 K. Blue atoms represent the body-centered cubic (BCC) structure, and white atoms represent the Other structure.

Figure 12 shows the internal atomic structural characteristics of the models under different hydrogen concentrations and temperatures after relaxation. Among these, OTHER structured atoms are those which, according to Common Neighbor Analysis (CNA), refer to atoms identified through this method that cannot be classified into any known long-range ordered crystal structure. By comparing the arrangement characteristics of the atoms under various conditions, it can be observed that at low temperatures, regardless of hydrogen concentration variations, the atomic arrangements within individual grains maintain high structural order, with the overall lattice structure remaining stable. It should be noted that at higher hydrogen concentrations (e.g., *C_H_* = 5%), although the model still maintains relatively neat atomic arrangements at low temperatures, the proportion of OTHER structured atoms increases dramatically when the temperature rises to 1200 K, and the disordered regions progressively extend from the grain boundaries into the grain interiors. This phenomenon signifies that hydrogen at elevated temperatures profoundly exacerbates the destructive effect of the lattice structure, which not only accelerates the atomic instability at grain boundaries but also promotes the inward diffusion of disordered structures into grain interiors, thus undermining the integrity and stability of the crystalline structure. In summary, at low temperatures, hydrogen concentration has a negligible effect on structural disorder, but under elevated temperatures, the increase in hydrogen concentration markedly intensifies the degree of structural disorder.

## 4. Conclusions

This study employs MD simulations to investigate the strength response and damage evolution of a polycrystalline iron model containing hydrogen segregation under triaxial tensile conditions. The principal findings are summarized as follows:

(1) As hydrogen concentration increases, tensile strength increases at the low concentration and decreases at the high concentration, indicating the existence of an extremum of tensile strength as a function of hydrogen concentration. For *C_H_* ≤ 1%, hydrogen atoms promote the formation of loop structures through dislocation entanglement, and increased dislocation resistance slows void growth. Consequently, the peak tensile stresses at *C_H_* ≤ 1% become notably higher than the peak tensile stress in the hydrogen-free case. However, as the hydrogen concentration continuously increases, the peak tensile stress gradually decreases and eventually falls below that under hydrogen-free conditions.

(2) The evolution of the void volume fraction exhibits a characteristic three stages: initial growth, rapid growth, and slow growth. At these stages, the processes of void nucleation, growth and coalescence occur, primarily in the grain boundary regions. Moreover, the evolution of void surface area in the model essentially results from the competition between two mechanisms: the decrease caused by void collapse and coalescence and the increase caused by void expansion. Additionally, it is found that voids first form around hydrogen-containing configurations and play a key role in the stability of the cluster structure; the growth and coalescence of voids around the clusters lead to severe distortion and a substantial reduction in their stability.

(3) Under high-temperature conditions, temperature plays a key role in determining strength, while the strengthening effect of low hydrogen concentrations can be neglected. Further analysis shows that at 300 K, all grains maintain a highly ordered structure, and variations in hydrogen concentration have minimal impact on structural ordering. In contrast, at elevated temperatures, the increase in hydrogen concentration markedly exacerbates the disorder of the crystal structure.

Although this study reveals the atomic-scale mechanisms of hydrogen-induced damage, it has certain applicability limitations. First, regarding the time scale and hydrogen transport assumptions, the high strain rate of 5 × 10^9^ s^−1^ applied in this simulation corresponds to extreme loading conditions such as laser loading experiments (where strain rates can reach the order of 10^9^ or higher), which are significantly higher than the quasi-static conditions in conventional experiments. This extremely short, nanosecond-scale loading time leads to two effects: (1) It results in numerically higher yield stresses compared to typical experimental values, and thermally activated void nucleation processes may be delayed. (2) The characteristic time of deformation is much shorter than the long-range diffusion time of hydrogen atoms. This means the simulation cannot capture the dynamic redistribution of hydrogen atoms during deformation (i.e., it cannot replicate the dynamic shielding effect under quasi-static conditions). However, the pre-segregation model adopted in this study reasonably circumvents this limitation by establishing a thermodynamic equilibrium distribution before loading. This model simulates a “worst-case scenario” where hydrogen has already aggregated at grain boundaries, effectively isolating diffusion kinetics and focusing on revealing the intrinsic mechanism by which hydrogen reduces interfacial cohesion. Second, regarding damage modeling and spatial scale, this study employs the EAM potential function for pure iron, which represents an idealized matrix model. Common features in macroscopic ferritic steels, such as carbides, non-metallic inclusions, and larger voids, are not explicitly included in the model. Therefore, this model reflects the inherent damage resistance of the ferritic matrix rather than the overall lifetime of engineering alloys. Despite the above limitations and the geometric differences between the nanocrystalline model and macroscopic coarse-grained structures, this study provides a crucial window into atomic-scale damage evolution. It successfully decouples complex macroscopic factors and elucidates the competitive mechanisms of hydrogen-induced grain boundary decohesion and dislocation pinning. These microscopic processes are the fundamental origins of damage initiation in macroscopic polycrystalline materials.

## Figures and Tables

**Table 1:** Void nucleation time at hydrogen concentrations ranging from 0% to 5%.

Hydrogen Concentration	0%	0.5%	1%	3%	5%
void nucleation time (ps)	11.8	12.2	12	11.6	11.4

## Data Availability

The original contributions presented in this study are included in the article. Further inquiries can be directed to the corresponding authors.
